# Compost Tea Combined with Fungicides Modulates Grapevine Bacteriome and Metabolome to Suppress Downy Mildew

**DOI:** 10.3390/jof11070527

**Published:** 2025-07-16

**Authors:** Giuliano Bonanomi, Giuseppina Iacomino, Ayoub Idbella, Giandomenico Amoroso, Alessia Staropoli, Andrea De Sio, Franco Saccocci, Ahmed M. Abd-ElGawad, Mauro Moreno, Mohamed Idbella

**Affiliations:** 1 Department of Agricultural Sciences, University of Naples Federico II, Via Università 100, 80055 Portici, Italy; giuliano.bonanomi@unina.it (G.B.); ayoub.id-bella@unina.it (A.I.); giandomenico.amoroso@unina.it (G.A.); andrea.desio3@gmail.com (A.D.S.); mauro.moreno@unina.it (M.M.); 2 Task Force on Microbiome Studies, University of Naples Federico II, 80100 Naples, Italy; 3 Department of Veterinary Medicine and Animal Productions, University of Naples Federico II, Via F. Delpino, 1, 80138 Naples, Italy; alessia.staropoli@unina.it; 4 Independent Researcher, 03020 Castro dei Volsci, Italy; saccocci.franco@gmail.com; 5 Plant Production Department, College of Food & Agriculture Sciences, King Saud University, P.O. Box 2460, Riyadh 11451, Saudi Arabia; aibrahim2@ksu.edu.sa; 6 AgroBioSciences (AgBS) Program, College of Agriculture and Environmental Sciences, Mohammed VI Polytechnic University, Ben Guerir 43150, Morocco; mohamed.idbella@um6p.ma

**Keywords:** compost, disease suppression, downy mildew, organic amendment, biostimulants

## Abstract

Downy mildew, caused by *Plasmopara viticola*, is a major threat to grapevine (*Vitis vinifera*) cultivation in humid climates. Restrictions on synthetic pesticides and inconsistent efficacy of current biocontrol agents, especially under rainy conditions, complicate disease management. This study evaluated the potential of compost tea to suppress downy mildew in a two-year field experiment (2023 and 2024), combined with reduced synthetic fungicide applications. The study design compared two phytosanitary management strategies on a commercial vineyard: a conventional fungicide against a compost tea strategy supplemented with two cymoxanil applications. The experiment set up had three replicated blocks, each consisting of 100 plants for a total of 600 plants. Mechanistic insights were provided through controlled laboratory experiments involving pre- and post-infection leaf assays, vineyard bacteriome profiling, via 16S rRNA gene sequencing for bacterial communities, across vineyard compartments, i.e., bulk soil, rhizosphere, and phyllosphere, and grapevine metabolomic analysis by GC-MS analysis. Field trials demonstrated that compost tea combined with two fungicide applications effectively reduced disease severity, notably outperforming the fungicide alone in the particularly rainy year of 2023. Bacteriome analysis revealed that compost tea treatment enriched beneficial bacterial genera, including *Pseudomonas*, *Sphingomonas*, *Enterobacter*, *Massilia*, and *Bacillus*, known for their growth-promoting and biocontrol activity in the rhizosphere and phyllosphere. Laboratory assays on detached leaves further showed that compost tea alone could suppress both infection and sporulation of *P. viticola.* Metabolomic analysis highlighted the accumulation of compounds such as tartaric and shikimic acids in compost tea treated leaves, suggesting a potential role in induced resistance. The findings indicate that applying compost tea with reduced fungicide treatments represents a promising and sustainable strategy for managing grapevine downy mildew, even in challenging climates.

## 1. Introduction

The cultivation of grapevines, covering approximately 7 million hectares worldwide, is one of the most economically significant agricultural activities globally [1]. Historically rooted in Europe, with Italy, France, and Spain as leading producers, grapevine cultivation has also expanded significantly to regions such as the United States, Australia, Chile, and South Africa. The grapevine (*Vitis vinifera* L.) is highly susceptible to various pathogens, with downy mildew, caused by the oomycete *Plasmopara viticola* Berl. & De Toni, being among the most devastating in warm, humid climates characterized by frequent rainfall during flowering and fruit set [2]. While resistant varieties have been developed through crossbreeding with American vine species, their adoption remains limited [3], necessitating reliance on careful and costly chemical pesticide applications to protect crops.

From 1890 until the end of the Second World War, the fight against downy mildew relied primarily on copper-based products, such as Bordeaux mixture, discovered serendipitously by Millardet in 1882 [4]. After the war, the introduction of multi-site synthetic fungicides with contact action, such as dithiocarbamates (e.g., maneb, mancozeb, metiram, propineb, thiram, ziram, and zineb), simplified disease management due to their lower phytotoxicity compared to copper. However, these products now face strict regulations because of their ecotoxicological impacts. In the 1980s, the diffusion on the market of fungicides with cytotropic and systemic capability like cymoxanil and metalaxyl, offering preventive and curative effects, revolutionized disease control. These products, still central to conventional and integrated agriculture, are prone to the development of resistance by pathogens due to their single-site mode of action [5]. Moreover, their environmental consequences, including soil and water contamination and potential entry into the food chain, have caused growing concern among consumers about the presence of fungicide residues in food products [6]. In organic agriculture, copper remains the cornerstone of downy mildew management. Although copper is not subject to the evolution of resistance by major pathogens, this heavy metal accumulates in the soil causing phytotoxicity and disrupting soil microbiomes and fauna. As a result, its use is quantitatively restricted and targeted for replacement within the European Union.

Significant research efforts have focused on alternative solutions to replace or complement copper. Biocontrol agents such as *Trichoderma harzianum* T39 [7], *Epicoccum nigrum* [8], *Fusarium proliferatum* [9], *Alternaria alternata* [10], *Lysobacter capsica* [11], and the hyperparasite *Trichothecium plasmoparae* [12] have been extensively studied. Resistance inducers like the non-protein amino acid BABA, chitosan, laminarin, and plant extracts have also received considerable attention [2,13]. However, these alternatives have seen limited commercial adoption due to their inconsistent efficacy, particularly during periods of high pathogen pressure. The unique autecology of *P. viticola*, which is exclusively associated with liquid films during the infection phase and may complete the penetration in just 2.5 h at 24 °C, likely provide one explanation for the ineffectiveness of fungal biocontrol agents. In fact, the majority of biocontrol fungi are still in the early stages of germination during *P. viticola* infection, indicating that a key component of successful biocontrol is the rate at which the fungus colonizes when wet leaves are present. The importance of developing new defense systems is emphasized by the recent epidemic of 2023 in Central and Southern Italy that experienced over 30 rainy days and more than 300 mm of rainfall during May and June, resulting in downy mildew-related losses of 50% to 100% on organic farms. These challenges highlight the urgent need for effective and reliable copper alternatives that can withstand adverse weather conditions, including frequent and heavy rainfall.

In this context, compost tea presents a promising strategy for suppressing plant pathogens. Compost tea is produced through the short-term aerobic or anaerobic brewing of various organic feedstocks in water and is well-known for its plant growth-promoting properties [14]. Both researchers and farmers have shown significant interest in its potential to suppress numerous plant diseases. In their seminal review, Scheuerell and Mahaffee [15] highlighted the ability of compost tea to control a range of foliar and soil-borne diseases caused by biotrophic and necrotrophic pathogens. Subsequent studies have reported its suppressive effects on pathogens such as *Pythium ultimum* [15] and *Rhizoctonia solani* [16], as well as foliar diseases caused by *Botrytis cinerea* and *Alternaria alternata* [17], *Phytophthora infestans* [18], and various powdery mildew species [19,20]. However, the suppressive effect of compost tea is variable, with studies reporting positive effects and others indicating limited efficacy and in others even an increase in the incidence of diseases. The lack of standardization in the raw feedstocks for the production of compost tea as well as the process conditions are elements that certainly contribute to the variability of the results reported in the literature. Surprisingly, despite this growing body of research, there are no published studies, aside from anecdotal reports in gray literature, on the suppressive effects of compost tea against grapevine downy mildew. This gap underscores the need for targeted research to explore its potential as a sustainable alternative for managing this economically significant disease.

The primary objective of this study is to assess the suppressive capacity of compost tea against grapevine downy mildew in an open vineyard setting when combined with synthetic fungicides currently employed to manage this disease. Additionally, the study investigates the mechanisms underlying the suppressive effects of compost tea through a combination of pre- and post-infection assays, extensive bacteriome characterization across vineyard compartments (soil, rhizosphere, and phyllosphere), and a metabolomic analysis to evaluate the grapevine’s physiological response to compost tea application. To ensure the stability and replicability of the results, a fully chemically and microbiologically characterized starting feedstock was used and the brewing conditions were defined both in terms of temperature and volume of oxygen supplied. The study focuses on the following specific hypotheses:

i. Compost tea reduces the severity and incidence of grapevine downy mildew in open vineyard conditions when integrated with synthetic fungicides;
ii. The suppressive effects of compost tea are mediated by the ability of its bacterial community to establish and persist within the rhizosphere and phyllosphere, enhancing the plant’s natural defense barriers;
iii. Compost tea application induces specific metabolomic changes in grapevines, including the upregulation of key metabolic pathways associated with enhanced resistance to *P. viticola*.

## 2. Materials and Methods

### 2.1. Compost Tea Preparation, Chemical and Microbiological Analysis

The commercial compost tea Stimol-C^®^, produced by GWA (GimaWater & Air S.r.l., Anagni, Italy), was utilized in this study. The solid organic feedstock comprised hay from polyphyte meadows (20%), compost from cow and horse manure and straw (30%) sourced from organic farms, and dried sunflower residues (50%). The organic fraction of the compost tea used in the study was recently analyzed by ^13^C CPAMS NMR [21]. Before the aerobic brewing process, standard techniques were employed to analyze the solid fraction’s properties, including water content, pH, organic carbon content, humic and fulvic acid concentrations, total nitrogen, C/N ratio, and electrical conductivity.

Compost tea was prepared in a 1000 L polyethylene, non-biodegradable container by mixing tap water with the organic feedstock at a ratio of 1:100 (*w*/*v*). The solid fraction, enclosed in a porous bag with a 0.2 mm mesh, was submerged in the water and brewed for 24 h. During the process the liquid was aerated with an air pump (Secoh Air Pump, model JDK-40, Wholesale Septic Supply, Dayton, TX, USA) that introduced 60 L of air per minute and generated very fine air bubbles. After the 24 h brewing period, compost tea was immediately applied to the plants for agronomic studies, without undergoing storage. Compost tea was freshly brewed every ten days and promptly sprayed on crops following preparation. The compost tea was applied at 600 L per hectare by means of a 3-point mounted sprayers model CIMA, sprayer New Plus 55 (CIMA SpA, Pavia, Italy).

The compost tea’s chemical properties, including pH, electrical conductivity (EC), dissolved organic carbon (DOC), dissolved organic nitrogen (DON), biological oxygen demand (BOD), dissolved oxygen, nitrate, and ammonium, were measured. Dissolved oxygen, EC, and pH were determined using a multi-parametric probe (M40+ instrument, Crison, Alella, Spain). The IRSA-CNR 5110 protocol was employed to analyze DOC, DON, and BOD, while the respirometric approach utilized the Oxitop OC100 system (OXITO-C) (WTW^®^ Xylem^®^ OxiTop^®^ OC100 Xylem Inc.301 Water Street SE, Suite 200, Washington, DC, USA). Nitrate and ammonium concentrations were assessed using a DR 3900 Spectrophotometer (Hach, Loveland, CO, USA) with manufacturer kits LCK 340 (assay range 5–35 mg L^−1^) for nitrate and LCK 303 (assay range 2–47 mg L^−1^) for ammonium. The chemical characteristics of compost tea at the time of applications are reported in Table 1.

Concerning compost tea safety, according to the official procedures established by the Italian Ministry of Agricultural Food and Forestry Policies (MIPAF 2014), the presence of human pathogens, specifically *Salmonella* spp. and *Escherichia coli*, has been evaluated on organic feedstock. In this regard, *Salmonella* spp. and *E. coli* were not detected in the solid feedstock by our systematic microbiological investigations.

### 2.2. Field Evaluation of Compost Tea for Managing Downy Mildew in Vineyards

The field experiment was conducted in the spring and summer of 2023 in an 18-year-old Montepulciano vineyard located in Cerignola, Puglia, Southern Italy, with a planting density of 2500 plants per hectare. All the viticultural practices, with the exception of disease control, including fertilization, irrigation, and pruning, were comparable to the standard agronomical management adopted in the surrounding area. The soil is poor in terms of organic carbon (1.4%), with pH of 7.8, EC of 220 μS cm^−1^, total nitrogen (N) content of 1.1 g kg^−1^, phosphorus pentoxide (P_2_O_5_) content of 2.8 mg kg^−1^, total limestone of 153.7 g kg^−1^, Magnesium of 0.2 g kg^−1^, exchangeable Sodium of 0.02 g kg^−1^, Potassium of 1.34 g kg^−1^, Iron of 43.2 mg kg^−1^, Copper of 22.0 mg kg^−1^, Zinc of 5.4 mg kg^−1^, and Manganese of 6.0 mg kg^−1^.

The objective was to test the effectiveness of compost tea in controlling downy mildew under field conditions. Two phytosanitary management strategies were compared. The first strategy involved conventional farm management, which relied on synthetic fungicides such as cymoxanil, metalaxyl, strobilurins, and zoxamide, applied every 10 to 14 days depending on climatic conditions. Due to frequent rains in 2023 [22], a total of 15 applications were made from mid-April to late July. The second strategy utilized compost tea, applied at a rate of 600 L per hectare every 10 to 14 days, supplemented by two cymoxanil applications during the post-flowering and fruit-setting phases. In total, 13 compost tea applications and two cymoxanil applications were performed. Notably, no copper-based products were used in either plant protection strategy. The fungicides were applied at the dosage reported on the label and applied by means of 3-point mounted sprayers (model CIMA, sprayer New Plus 55).

The experiment was set up with three replicates per strategy, with each replicate consisting of 100 plants arranged across four vineyard rows for a total of 600 plants. At the end of July 2023 and 2024, the severity of downy mildew on both leaves and grape bunches was visually assessed. In each block, 20 plants were randomly selected, and data were collected on the number and size of downy mildew spots on leaves, as well as the presence and extent of infection on grape bunches. Statistical analysis of the differences between the two defense strategies was performed using a *t*-test.

### 2.3. Grapevine and Compost Tea Bacteriome

The objective of this activity was twofold: first, to characterize the bacterial community of the compost tea itself, and second, to compare the bacteriome of the vineyard ecosystem’s main compartments, soil, rhizosphere, and phyllosphere, under treatments with either compost tea or synthetic fungicides. In late July 2023, samples were collected for analysis. Freshly brewed compost tea was sampled by taking 1 L at the end of the brewing process conducted on the farm. For the vineyard bacteriome, 50 fully expanded, non-senescent leaves were collected from each of the 20 plants previously selected for downy mildew assessment. Bulk soil samples were taken from five locations within each block, combined to form one composite sample per block. Soil was collected from the top 30 cm of the profile after removing surface litter and weeds. Rhizosphere samples were obtained by carefully excavating soil to expose vine roots and collecting the soil adhering to them. All samples, phyllosphere, rhizosphere, and bulk soil, were placed in polyethylene bags, kept refrigerated at 4 °C during transport to the laboratory on the same day, and subsequently stored at −20 °C until DNA extraction.

DNA was extracted from the collected samples, including compost tea, bulk soil, rhizosphere soil, and phyllosphere, using the DNeasy PowerSoil Kit (Qiagen, Germantown, MD, USA) according to the manufacturer’s protocol. For compost tea, 500 mL of the sample was filtered through a 0.2 μm membrane to concentrate bacterial cells. The membrane was cut into small pieces and processed directly using the PowerSoil kit. For bulk soil samples, 0.25 g of soil was used for each extraction, ensuring representative sampling. Rhizosphere samples were carefully prepared by processing only the soil tightly adhering to the vine roots. For the phyllosphere, bacterial cells were detached by washing leaves in sterile phosphate-buffered saline by vortexing for 5 min. The wash solution was filtered through a 0.2 μm membrane, which was then processed with the PowerSoil kit. The quantity of extracted DNA was measured using a NanoDrop 2000 spectrophotometer (Thermo Fisher Scientific, Waltham, MA, USA), and quality was verified via agarose gel electrophoresis. PCR amplification targeted the V3–V4 region of the 16S rRNA gene (approximately 460 bp) to profile bacterial communities. Bacterial 16S rRNA amplification was performed using primers S-D-Bact-0341-b-S-17 and S-D-Bact-0785-a-A-21. Amplification conditions followed the protocols described in the respective studies. PCR products were purified using Agencourt AMPure XP beads (Beckman Coulter, Milan, Italy) and quantified with an AF2200 Plate Reader (Eppendorf, Milan, Italy). Libraries were prepared according to Illumina’s standard protocols, and sequencing was performed on the MiSeq platform (Illumina, Milan, Italy), generating paired end reads of 2 × 250 bp.

### 2.4. Effects of Pre- and Post-Infection Applications of Compost Tea on P. viticola

In order to further understand the suppressive effect of compost tea on downy mildew observed in open fields, two experiments were conducted in spring 2024 under controlled conditions to investigate the potential effect on the infection and sporulation process of *P. viticola.* The experiments were performed using whole leaves in one case and leaf disk in the second case in the laboratory. Briefly, three-year-old plants of the *V. vinifera* cv. Montepulciano were grown under shade-greenhouse conditions in large, 30 L pots. A *P. viticola* population was collected in a vineyard not treated located in the Department of Agriculture in May 2024 and maintained by periodical inoculations on potted grapevine grown in shade-house. The *P. viticola* inoculum was prepared collecting disease leaf with evident symptoms that were incubated overnight in darkness at 100% RH and 22 ± 2 °C to promote the sporulation of the pathogen. Sporangia were then collected by gently washing the abaxial leaf surfaces bearing freshly sporulating lesions with distilled water. The inoculum concentration was adjusted to 1 × 10^5^ sporangia mL^−1^ by light microscope using a hemocytometer. The sporangia suspension, in the experiments, was applied to the abaxial leaf until the surface was completely wet. For the inoculum, grape leaves collected from *V. vinifera* cv. Montepulciano were surface sterilized by immerging them for 1 min in 1% *v*/*v* solution of sodium hypochlorite. After that, the leaves were abundantly rinsed three times with distilled water and dried on filter paper. Entire excised leaf or leaf disk with a 15 mm diameter were cut using a scissor and placed, abaxial side up, in Petri dishes (9 cm), over moistened filter paper layer. After the inoculum, Petri dishes were sealed with parafilm and incubated in a growth chamber at 24 ± 2 °C in the dark. Disease severity was quantified at 5 days after inoculation (dpi) as percentage of leaf area that was covered by *P. viticola* lesion and sporulation. Twenty replicates were assessed for each treatment and each experiment was carried out twice.

In the first experiment, aimed at investigating the infection process, entire and excised grapevine leaves prepared as described above were sprayed with compost tea or distilled water as control and left to dry for 2 h. Following this treatment, the leaves were inoculated with sporangia as described above and incubated in dark conditions at 22 ± 2 °C, with disease severity quantified after 5 days of dpi. In the second experiment, aimed at investigating the effect on sporulation, infected grapevine leaves with evident sporulation on the abaxial leaf surface were sampled. Leaf disks were cut with scissors at the points of abundant sporulation and emerged in the previous 24 h. These leaf disks were sprayed with compost tea or distilled water and subsequently incubated as described. After two days the state of the sporulated areas was evaluated using an optical microscope.

### 2.5. Metabolomics of Grapevine Leaves Treated with Compost Tea

For metabolomic analyses, grape leaves were collected from *Vitis vinifera* cv. Montepulciano grown in greenhouses at the Department of Agriculture, located in the Royal Park of Portici (40°48′40.3″ N, 14°20′33.8″ E; 75 m a.s.l.). The leaves were categorized into four groups: (1) healthy grapevine leaves not treated with compost tea (water), (2) healthy grapevine leaves treated with compost tea (compost tea), (3) grapevine leaves affected by downy mildew but not treated with compost tea (water inoculated), and (4) grapevine leaves affected by downy mildew and treated with compost tea (compost tea inoculated). The plant material was freeze-dried (Zirbus VaCo 2, ZIRBUS Technology GmbH, Bad Grund, Germany) and ground using a mortar and pestle. For each sample, 50 mg of the powdered material was extracted using three sequential solvents: methanol, dichloromethane, and n-hexane (500 µL each). After the addition of each solvent, the suspension was vortexed for 30 s and centrifuged at 13,000 rpm for 10 min. The supernatants were pooled and evaporated to dryness using a vacuum concentrator (Savant™ SpeedVac™ SPD130, Thermo Fisher Scientific Inc., USA). The dried extracts were derivatized by adding 1 mL of N,O-bis(trimethylsilyl)trifluoroacetamide (BSTFA, Merck KGaA, Darmstadt, Germany). The reaction mixture was incubated in an ultrasonic bath (Sonorex, Bandelin Electronic GmbH & Co. KG, Berlin, Germany) at room temperature for 30 min.

### 2.6. GC-MS Analysis

The trimethylsilyl derivatives were analyzed using an Agilent 8890 GC system (Agilent Technologies, Santa Clara, CA, USA) coupled with an Agilent 5977B Inert MS detector. The instrument was equipped with an HP-5MS capillary column (5%-phenyl)-methylpolysiloxane stationary phase) for compound separation. The GC oven was programmed with the following temperature gradient: an initial temperature of 90 °C, increasing at a rate of 10 °C/min to a final temperature of 300 °C, which was held for 10 min. The solvent delay was set to 5 min. The injector operated in splitless mode at 250 °C, and helium was used as the carrier gas at a flow rate of 1 mL/min. The injection volume was 1 µL. Mass spectrometry measurements were performed in full scan mode (*m*/*z* 35–550) with electron impact (EI) ionization at 70 eV. The ion source and quadrupole mass filter temperatures were maintained at 230 °C and 150 °C, respectively. Metabolite identification was performed by comparing the acquired mass spectra with those stored in the NIST20 library. Identification was considered successful when the match factor associated with the comparison was above 800.

### 2.7. Data Processing and Statistical Analysis

Metabolomic statistical analysis was performed using Mass Profile Professional software, version 13.1.1 (Agilent Technologies). Raw data were grouped by experimental condition and subjected to univariate analysis between paired conditions using Student’s *t*-test (*p*-value < 0.05), with a fold change threshold of ≥2.0. Metabolite identification was achieved by comparing the obtained mass spectra with those in the NIST20 library (National Institute of Standards and Technology). Aligned abundance values were exported to R for further analysis. Principal Coordinates Analysis (PCoA) was conducted using the vegan package (function capscale) to explore relationships between experimental conditions based on Spearman rank correlations. The results were visualized as a 2D scatter plot using the ggplot2 package. Diversity indices were calculated using the vegan package (function diversity), and box plots representing the distribution of the indices were generated with ggplot2. Hierarchical clustering analysis was performed using the pheatmap package to generate a heatmap of metabolite relative abundance. Clustering was performed using the hclust function with Euclidean distance and complete linkage. Venn diagrams to compare the overlap of identified metabolites across different experimental conditions were created using the VennDiagram package, specifically with the venn.diagram function.

For bacterial community composition analysis, relative abundance data were visualized as stacked bar plots using ggplot2. PCoA was performed on Bray–Curtis dissimilarity matrices using the vegan package (functions vegdist for distance calculation and ordinate for ordination). The resulting plot was created using ggplot2. Differences between groups were assessed using PERMANOVA (function adonis from the vegan package) with 999 permutations. Alpha diversity metrics, including species richness (observed ASVs), Shannon index, and Pielou’s evenness, were calculated using the vegan package (functions specaccum for species richness and diversity for Shannon index and Pielou’s evenness). Boxplots were generated using ggplot2, and statistical significance between experimental groups was determined using one-way ANOVA followed by post hoc Tukey’s HSD test for multiple comparisons. The Venn diagram for bacterial taxa overlap was created using the limma package (function voom for variance modeling and differential abundance analysis), with the overlap visualized using the Venn function from limma to highlight shared and unique ASVs between the experimental groups.

## 3. Results

### 3.1. Field Evaluation of Compost Tea for Controlling Downy Mildew

**Figure 1:**
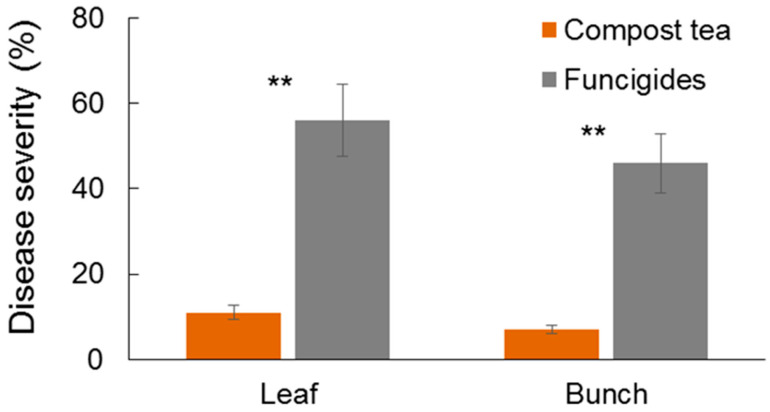
Severity of downy mildew observed on leaves and bunches in July 2023 in a vineyard (cv. Montepulciano). Treatments included compost tea combined with cymoxanil and a standard synthetic plant protection management approach (fungicides). Values represent the mean of three replicates, with error bars indicating standard deviation. Statistical comparisons between treatments were performed using independent two-sample *t*-tests. Asterisks (**) indicate highly significant differences between treatments (*p* < 0.01).

To assess the efficacy of compost tea in controlling downy mildew under real vineyard conditions, a two-year field trial was conducted in 2023 and 2024. This experiment compared a compost tea-based management strategy with a conventional synthetic fungicide program, evaluating disease severity on leaves and grape bunches. The severity of the disease was significantly lower in plants treated with compost tea compared to those managed with synthetic fungicides in 2023, for both leaves and bunches (Figure 1). In 2024, however, the environmental conditions were not favorable for disease development, resulting in disease severity of 4.1% and 4.9% in the plots treated with synthetic fungicides and in those treated with compost tea, respectively, with no significant differences observed (*t*-test, *p* = 0.78). The high incidence and severity detected in 2023 compared to 2024 is associated with the particularly rainy climate in the months of May and June with over 30 rainy days and more than 300 mm of rainfall. This difference between years underscores the impact of environmental variability inherent in field studies; the high incidence and severity detected in 2023 compared to 2024 is associated with the particularly rainy climate in the months of May and June, featuring over 30 rainy days and more than 300 mm of rainfall. Despite the lower disease pressure in 2024, the 2023 results provide robust evidence of compost tea’s efficacy under conditions highly conducive to downy mildew.

### 3.2. Impact of Compost Tea on Grapevine Bacteriome

**Figure 2:**
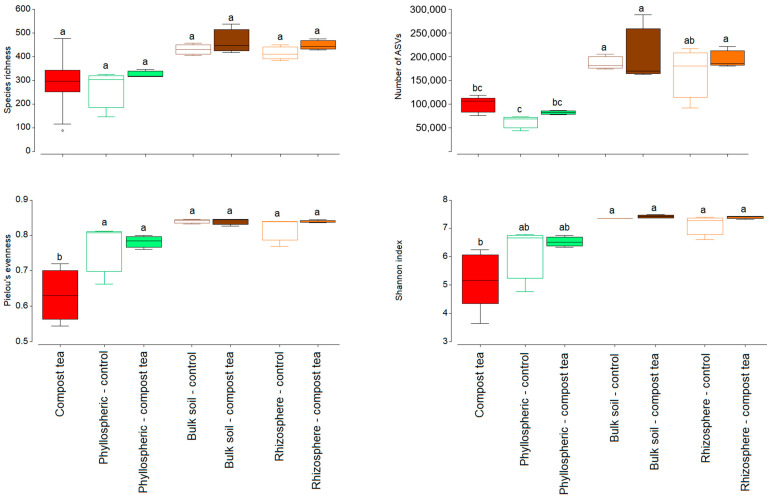
Species richness and diversity of bacterial communities in compost tea and the phyllosphere, bulk soil, and rhizosphere of grapevines treated or untreated with compost tea. Values represent the means of four replicates. In each boxplot, the lower and upper edges represent the first and third quartiles (25th and 75th percentiles), the horizontal line within the box indicates the median, and whiskers extend to the interquartile range. Statistical differences among groups were assessed using one-way ANOVA followed by Tukey’s Honest Significant Difference (HSD) post hoc test. Different letters above the boxes indicate statistically significant differences between groups (*p* < 0.05).

To investigate the bacterial mechanisms underlying compost tea’s suppressive effects, the bacterial communities of the compost tea itself and the main grapevine ecosystem compartments (bulk soil, rhizosphere, and phyllosphere) were profiled. Our results show that bacterial diversity metrics, including species richness, ASVs, Pielou’s evenness, and Shannon index, varied across treatments and compartments (Figure 2). Species richness was highest in the bulk soil under compost tea treatment, whereas the phyllosphere, bulk soil, and rhizosphere under both control and compost tea treatments exhibited comparable levels of richness without significant differences. The number of ASVs was highest in the rhizosphere under compost tea treatment, followed by the bulk soil under the same treatment, while compost tea alone, phyllosphere, and phyllosphere under compost tea displayed a lower number of ASVs. Pielou’s evenness was lowest in the compost tea treatment alone, significantly differing from all other treatments, which displayed similar levels of evenness across the compartments. Similarly, the Shannon index, which reflects both richness and evenness, was also lowest in the compost tea treatment alone, whereas the phyllosphere, bulk soil, and rhizosphere compartments under both control and compost tea treatments exhibited higher and comparable diversity levels.

**Figure 3:**
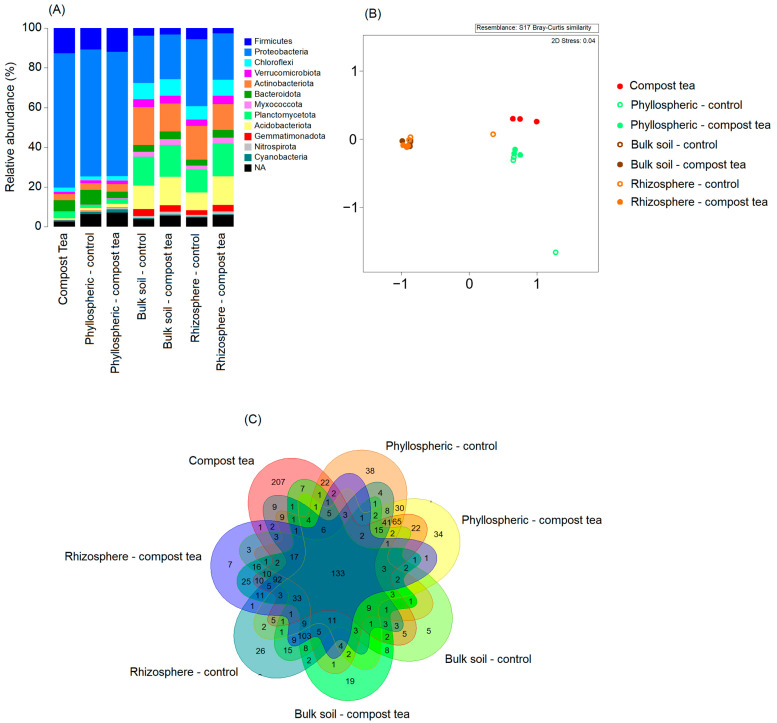
(**A**) Relative abundance of bacterial phyla detected in compost tea and in the phyllosphere, bulk soil, and rhizosphere of grapevines treated or untreated with compost tea. (**B**) Non-metric multidimensional scaling (NMDS) plot based on Bray–Curtis dissimilarity, showing bacterial community structure across different applied treatments. Each point represents a replicate sample. The two axes, MDS1 and MDS2, represent dimensions of the ordination space. The stress value (0.04) indicates a good fit of the data in two-dimensional space (values closer to 0 indicate a better representation of the original distance relationships). (**C**) Venn diagram depicting the number of ASVs unique to or shared among the applied treatments. Statistical analyses of community composition were conducted using PERMANOVA (permutational multivariate analysis of variance) on Bray–Curtis distances to assess significant differences among groups (*p* < 0.05).

Our results reveal distinct patterns in the bacterial community composition, structure, and shared taxa across treatments and compartments (Figure 3). The relative abundances of major bacterial phyla exhibit noticeable variations among treatments (Figure 3A). In the compost tea treatment, Proteobacteria dominate, constituting approximately 65% of the community, followed by Firmicutes at around 15%. Similarly, the phyllosphere compartments under both control and compost tea treatments show comparable distributions, with Proteobacteria comprising approximately 60% and Firmicutes around 10%. In contrast, the bulk soil and rhizosphere compartments display more diverse distributions, with Proteobacteria contributing 20–30%, Actinobacteriota 15–20%, Planctomycetota around 10–15%, and Acidobacteriota approximately 10–12%. Non-metric multidimensional scaling (nMDS) analysis based on Bray–Curtis similarity underscores the distinct separation of bacterial communities among the treatments (Figure 3B). Compost tea-treated samples form a unique cluster, while bacterial communities in the phyllosphere compartments exhibit close grouping regardless of treatment. In contrast, bulk soil and rhizosphere compartments cluster together, distinctly separated from the other compartments. The Venn diagram further highlights the distribution of unique and shared taxa across treatments (Figure 3C). A core set of 133 taxa is shared among all treatments. However, compost tea-treated samples harbor the highest number of exclusive taxa (207), followed by the phyllosphere under control (38) and compost tea (34) treatments. Conversely, bulk soil without compost tea shows the lowest number of exclusive taxa, with only four unique taxa identified.

**Figure 4:**
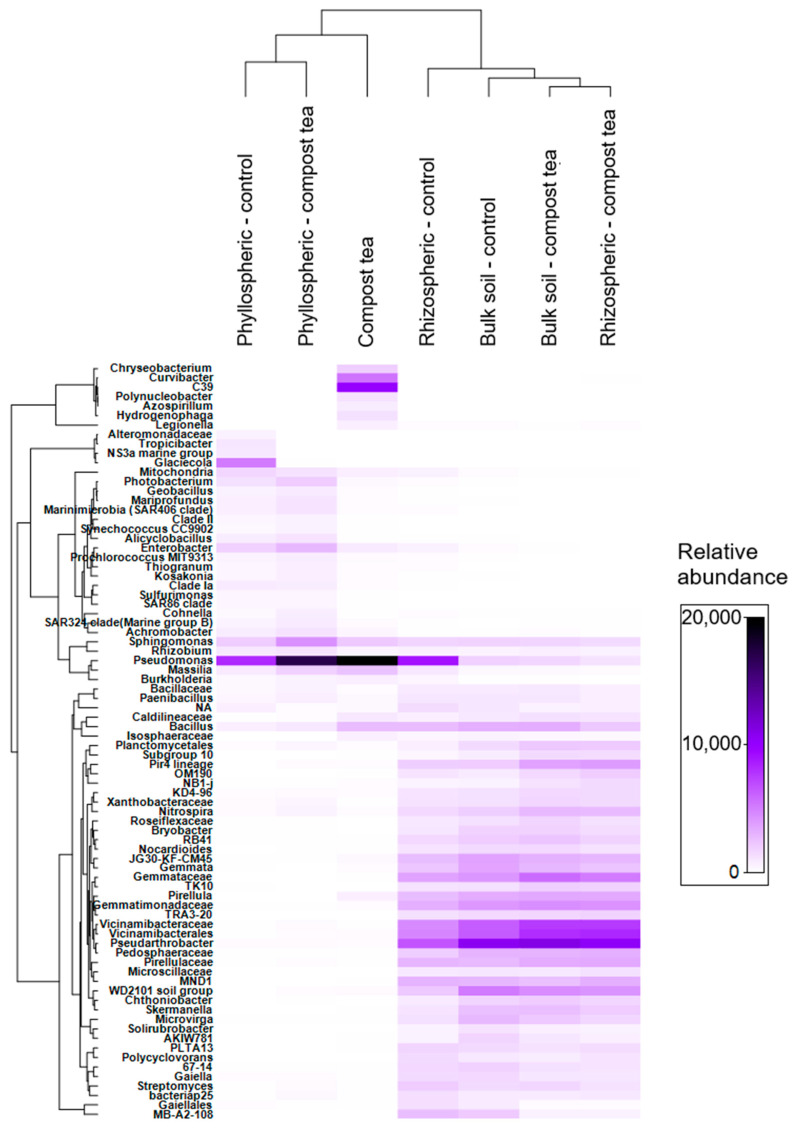
Heatmap showing a relative abundance of the 100 most frequent ASVs in the bacterial community in the different treatments. Hierarchical clustering of samples (columns) was performed using Bray–Curtis dissimilarity, while clustering of ASVs (rows) was based on Whittaker’s association index. The color gradient represents relative abundance values, with darker shades indicating higher abundance.

The hierarchical clustering of samples (Figure 4) reveals distinct grouping patterns that reflect bacterial community differences across compartments and treatments. Notably, compost tea alone is closely clustered with the phyllosphere microbiota, irrespective of the treatment condition, while rhizosphere samples are grouped alongside bulk soil samples. The heatmap also reveals significant differences in taxa abundance across compartments. The rhizosphere and bulk soil show a higher relative abundance of specific taxa, including Azotobacter, Pseudoarthrobacter, Vicinamibacterales, Gemmata, and Bacillus. In contrast, the phyllosphere exhibits a greater abundance of Pseudomonas and Sphingomonas. Pseudomonas are also dominant in compost tea. Additionally, Tuberibacillus is notably enriched in the phyllosphere. Interestingly, compost tea alone harbors unique taxa with relatively high abundance, such as Curvibacter, Azospirillum, and Cupriavidus necator C39.

### 3.3. Effect of Compost Tea on P. viticola Infection and Sporulation

**Figure 5:**
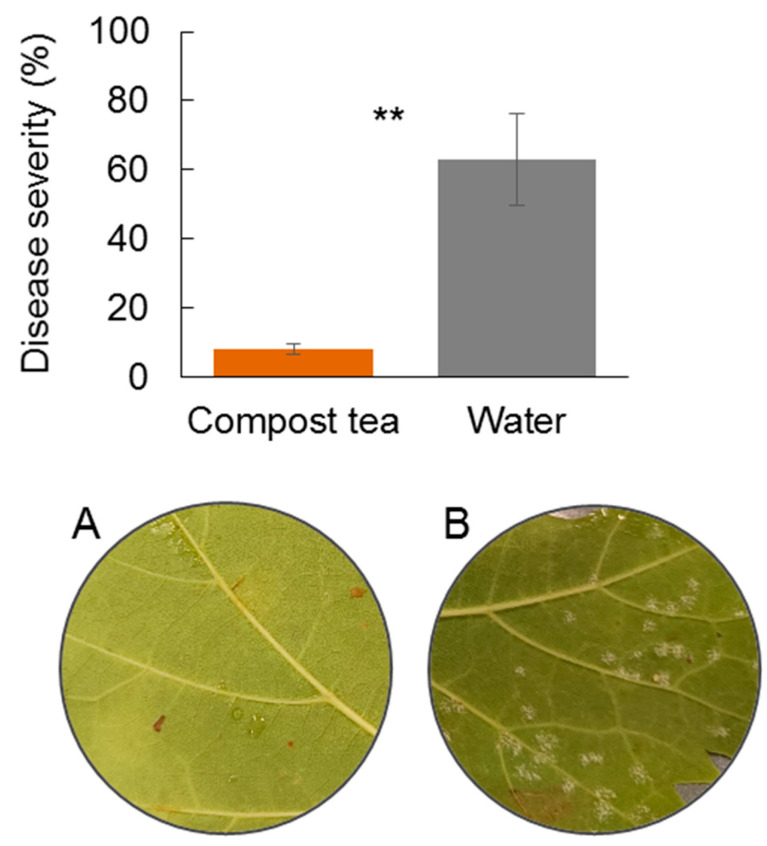
Severity of downy mildew detected on grapevine leaves (cv. Montepulciano) detached in the laboratory and treated with compost tea or water. Asterisks indicate highly significant differences (*t*-test, *p* < 0.01). Images of the abaxial leaf disk with little or no sporulation when treated with compost tea (**A**), or abundant sporulation in the control with water (**B**).

**Figure 6:**
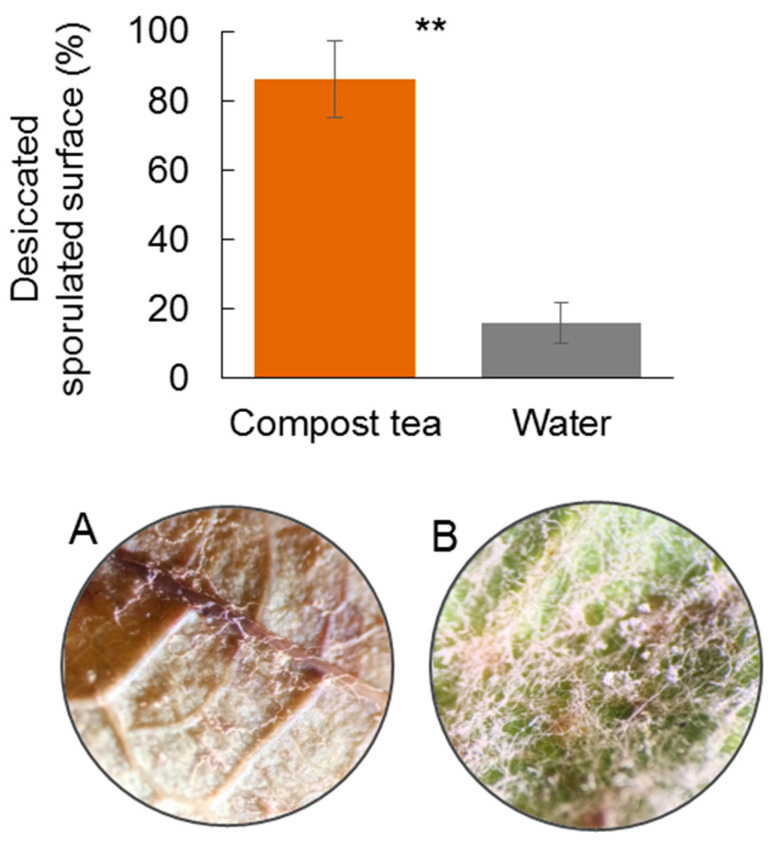
State of *P. viticola* sporangia forty-eight hours after treatment with compost tea or water. Asterisks indicate highly significant differences (*t*-test, *p* < 0.01). Images of the abaxial leaf disk with yellowed and dried sporangia when treated with compost tea (**A**), and sporulated area still white when treated with water (**B**).

To clarify the direct suppressive action of compost tea on the pathogen, controlled laboratory experiments assessed its effect on *P. viticola* infection and sporulation on grapevine leaves. In the first experiment with grapevine leaf, the application of compost tea drastically reduced downy mildew infection, compared to the water-treated control. The differences between the two treatments were different both three and six days after inoculation (Figure 5). In the second experiment with leaf disk, the application of compost tea on sporulated spots rapidly reduced their vitality. In fact, after only two days the sporangia appeared yellow and dried, suggesting a loss of functionality. On the contrary, the leaves treated with water showed abundant, white and not dried sporangia even after 5 days of incubation (Figure 6).

### 3.4. Impact of Compost Tea on Grapevine Metabolome

**Figure 7:**
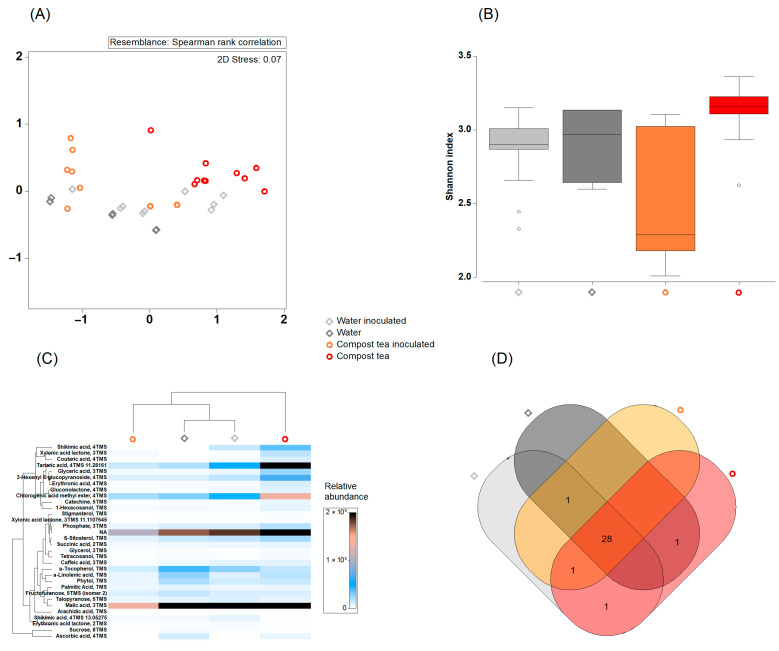
Panel (**A**) presents a non-metric multidimensional scaling (nMDS) plot based on Spearman rank correlation, highlighting the separation of samples by treatment groups: water inoculated, water, compost tea inoculated, and compost tea. Panel (**B**) shows box plots comparing the Shannon diversity index across treatments, indicating variation in microbial diversity. Panel (**C**) depicts a heatmap of relative identified metabolite abundances clustered by condition, with distinct profiles for each condition. Panel (**D**) presents a Venn diagram, showing the shared and unique metabolites across the four treatments. Statistical differences in alpha diversity (Shannon index) were assessed using one-way ANOVA followed by Tukey’s HSD post hoc test (*p* < 0.05). Multivariate differences in community composition were tested using PERMANOVA based on Spearman distances.

To elucidate the plant’s metabolic responses to compost tea and pathogen challenge, a comprehensive metabolomic analysis of grapevine leaves was performed. GC-MS analysis revealed significant alterations in metabolite profiles across the different conditions, with compost tea influencing the abundance of a wide range of metabolites, either increasing or decreasing their levels (Figure 7). The nMDS plot based on Spearman’s rank correlation (Figure 7A) illustrates the distinct grouping of grapevine leaf metabolite compositions under the four treatments. A clear separation is observed between inoculated and non-inoculated samples, particularly between compost tea-treated conditions compared to water. Furthermore, the boxplot representing the metabolite diversity index (Figure 7B) highlights differences among the treatments. Untreated healthy leaves (distilled water) and downy mildew-infected leaves without treatment (water inoculated) exhibit relatively high metabolite diversity, with no significant differences between these conditions. In contrast, downy mildew-infected leaves treated with compost tea (compost tea inoculated) show reduced metabolite diversity accompanied by greater variability. Interestingly, healthy leaves treated with compost tea (compost tea) display the highest median metabolite diversity. The heatmap (Figure 7C) illustrates the relative abundance of various metabolites across the four treatments. Hierarchical clustering distinguishes metabolite profiles into two major branches: one encompassing the healthy leaf treatments (gray) and the other grouping the downy mildew-infected leaf treatments (orange and red). Notably, several metabolites, such as tartaric acid, chlorogenic acid methyl ester, shikimic acid, and 3-hexenyl-β-glucopyranoside, are more abundant in compost tea-treated leaves, particularly those affected by downy mildew. Additionally, malic acid is detected across all treatments but shows higher abundance in compost tea-treated leaves. The Venn diagram (Figure 7D) represents the shared and unique metabolites found in grapevine leaves across the four treatments. The diagram shows 28 metabolites shared among all treatments, while some taxa are unique to specific conditions.

Table S1 presents the results of gas chromatography–mass spectrometry (GC-MS) analysis comparing the metabolic profiles in the four experimental conditions. In detail, in the comparison between downy mildew-affected grapevine leaves treated with water and healthy grapevine leaves treated with water, a significant decrease was observed only for α-Linolenic acid, indicating a reduction of this compound in the diseased leaves. The comparison between downy mildew-affected grapevine leaves inoculated with compost tea and healthy grapevine leaves treated with compost tea revealed several significant decreases, including glyceric acid, erythronic acid, and xylonic acid lactone. However, there was also a notable increase in 3-Hexenyl β-D-glucopyranoside, suggesting a potential response to the compost tea inoculation. In the comparison between healthy grapevine leaves treated with compost tea and those treated with water, significant increases were detected for several metabolites, such as glyceric acid and xylonic acid lactone, indicating a higher abundance of these compounds in response to the addition of compost tea. Some metabolites, like tetrahydroxycoumarin, showed a decrease in abundance. Finally, the comparison between downy mildew-affected grapevine leaves treated with compost tea and those treated with water revealed both increases and decreases in metabolite levels. Xylonic acid lactone, for instance, showed a marked increase, while succinic acid exhibited a significant reduction.

## 4. Discussion

Compost tea was effective, in combination with two applications of cymoxanil, in reducing the disease severity in field trial carried out in a commercial farm. The result is of particular interest for two reasons: first the use of compost tea was a superior defense strategy than the use of synthetic pesticides only, allowing a better control of downy mildew and saving 13 applications of fungicides. Second, the result was obtained in a climatically favorable year to the downy mildew with particularly rainy May and June. Our decision to conduct this study primarily under real vineyard conditions, rather than in controlled synthetic or greenhouse environments, was driven by the objective of evaluating compost tea’s efficacy and practical applicability in a representative agricultural setting. While controlled conditions provide valuable insights into specific mechanisms, they often do not fully replicate the complex environmental variability and intricate biological interactions characteristic of a real vineyard ecosystem. Therefore, our two-year field trial was specifically designed to assess real-world performance. The controlled laboratory assays on detached leaves served as a crucial complement to these field observations, allowing us to gain mechanistic insights into the direct suppressive effects of compost tea on pathogen infection and sporulation under defined conditions. From the point of view of effectiveness, the field results are very promising, but, undoubtedly, they require repetition in different production contexts and in years with different climate and grapevine variety before deserving a wider application. In addition, future studies will also have to compare the only use of compost tea or in combination with copper-based products to evaluate the possibility of defining new defense strategies for organic viticulture.

### 4.1. Impact of Compost Tea on Grapevine Microbiome and Disease Suppression

The study of the mechanisms underlying the suppressive compost tea are, however, equally important to make this product a real alternative to synthetic pesticides [14]. From this perspective our study provides a significant contribution, first revealing the impact of the tea compost on the microbiome and explaining how this interferes with two fundamental phases of the downy mildew cycle: infection and sporulation. Concerning microbiome, the compost showed a high bacterial species richness, but the diversity was lower compared to soil and rhizosphere because it was largely dominated by microbial groups within the *Proteobacteria* phylum, such as *Pseudomonas*, *Bacilllus*, *Massilla*, and *Sphingomonas.* One of the interesting findings of this study is the relative uniformity of the microbiome recorded across four distinct brewing processes carried out at farm scale. The variability in compost tea composition has long been cited as a major drawback, leading to uncertainty about its efficacy and reliability [23]. However, our results challenge this view by reporting that starting from a chemically balanced and standardized feedstock and controlling the brewing duration and oxygenation intensity can yield compost tea with consistent and relatively stable microbiome. This study has described the bacterial composition of the compost tea as a first, yet fundamental, step to understand their impact on crops and orchards; only a few studies have been conducted in this direction [24,25]. Our investigation, however, has also tried to understand if the bacteria present in compost tea could change the vineyard microbiome. In agreement with previous studies [26,27], our analysis showed that the different compartments of the vineyard have well defined microbiomes, with the rhizosphere and the bulk soil having greater species richness and diversity while the phyllosphere was dominated by proteobacteria. This is consistent with previous studies carried out in vineyards in different continents [28,29] and determined by the greatest stress in terms of fluctuations in terms of fear, moisture, exposure to ultraviolet rays, and reduced and floating availability of organic carbon in the phyllosphere compared to the soil. In this broad context, the repeated application of compost tea provides a periodic supply of beneficial microorganisms leading to changes in the phyllosphere and rhizosphere microbiome but without altering their essential characteristics. In the case of the phyllosphere, compost tea determines a slight but not significant increase in species richness and in the number of ASVs, while more relevant is the increase in some bacterial groups such as *Pseudomonas*, *Sphingomonas*, *Enterobacter*, *Massilla*, and *Bacillus* compared to the control treated with fungicides only. These bacterial groups include numerous species capable of stimulating plant growth [21,30,31] as well as biocontrol action against pathogens such as *B. cinerea* [32]. Their enrichment in the phyllosphere of treated plants suggests their possible role in suppressing downy mildew, a hypothesis that undoubtedly deserves further investigation. The effect of compost tea is not limited to the phyllosphere alone, with an evident impact also on the soil compartment. In fact, the application of compost tea determines changes in the telluric microbiome such that the rhizosphere and the bulk soil of the treated plants are more similar to each other than to the untreated samples that in the multivariate analysis cluster separately. It is interesting to note that the differences are determined by the enrichment of bacterial groups different from those enriched in the phyllosphere and not necessarily abundant in compost tea. For example, in the soils of the treated plants, there is a specific increase of Nitrospira, *Gemmataceae*, *Pseudarthrobacter*, but *Vicinamibacterales* and *Vicinamibacteraceae* especially are detected. *Vicinamibacterales* and *Vicinamibacteraceae* are particularly known to be common in grapevines grown in sandy soil [33]. The enrichment of these microbial groups in the soil by compost teas could be an indirect effect mediated by the supply of dissolved organic carbon present in the product that provides growth substrate that is added to the root exudates of the grapevine and to the stable fraction of carbon present in the soil. Future studies could investigate the possible beneficial effect of these microbial groups, for example, by inducing resistance in the grapevine to attacks by foliar pathogens.

In order to clarify the reasons underlying the suppressiveness of compost tea, laboratory experiments were conducted to investigate the effect on infection and sporulation of the pathogen. In fact, compost tea was effective in reducing both infection and sporulation on leaf disks and leaves of Montepulciano grapevine. This result suggests that compost tea can effectively interfere with two fundamental phases of the downy mildew cycle [3], on the one hand limiting infections and on the other reducing sporulation and therefore the development of secondary infections. The study, however, does not allow to identify which mechanisms allow compost tea to reduce infection and sporulation of *P. viticola.* Several studies, mainly in vitro with leaf disks, have demonstrated the ability of biocontrol agents to reduce the infection and sporulation of *P. viticola*. For example, spray application of *F. proliferatum* conidia suspension was effective pre- and post-infection and the authors observed that *P. viticola* sporangia was coiled and invaded by the mycelium of the biocontrol agent [9]. The hyperparasite *T. plasmoparae* effectively inhibited sporangial germination [12], while *A. alternata* reduced the sporulation, probably thanks to the production of some toxins [10]. Notably, the proteobacterium *Erwinia herbicola* is able to inhibit infection and sporangia germination, as well as alter the behavior of zoospores [2]. Despite the good results obtained in vitro, in some cases corroborated and supported by encouraging field trials for several years as for *F. proliferatum* [9], none of these biocontrol agents have been successfully introduced and distributed on the market. For example, despite the notable commercial successes of *Trichoderma* spp. in various herbaceous, horticultural, and arboreal crops [34], the application for the control of downy mildew was effective only in vitro [35]. A possible explanation could be linked to the particular autecology of *P. viticola* which, in the infection phase and when strictly linked to liquid films and in optimal conditions (about 24° and leaf wetness), is able to complete encasement and penetration in just 2.5 h. Most of the biocontrol fungi in such a time are still in the early stages of germination, suggesting that the speed of colonization in the presence of wet leaves is a fundamental element for effective biocontrol. In this context, compost tea with its high bacterial diversity could contain different species and therefore a pool of functions that generate its suppressiveness towards the pathogen. Compost tea can be assimilated into a microbial consortium with high diversity and, thanks to the particular production system, at low cost compared to products based on selected strains.

### 4.2. Compost Tea Induced Changes in Grapevine Metabolome

The metabolomic analysis highlights the significant influence of compost tea on both microbial communities and the metabolic profiles of grapevine leaves. Compost tea-treated grapevine leaves, whether healthy or infected, show distinct metabolic profiles compared to untreated leaves. Infected leaves treated with compost tea display more variability in microbial diversity, with lower levels overall. This indicates that compost tea might encourage a more specialized microbial community, potentially in response to pathogen pressure. Treated leaves also show an increase in defense-related metabolites, such as shikimic acid, a precursor in the phenylpropanoid pathway, which leads to the production of phenolic compounds that strengthen cell walls and inhibit pathogen growth [36]. This suggests that compost tea activates plant defense mechanisms, helping the plant resist pathogen attacks. Another notable metabolite influenced by compost tea is tartaric acid, a key organic acid in grapevine metabolism [37], crucial for grape quality and involved in the plant’s stress response. Healthy leaves treated with compost tea show a significant increase in tartaric acid levels, indicating that the treatment supports both normal metabolic functions and plant vigor. Interestingly, infected leaves typically exhibit reduced levels of tartaric acid, likely due to the metabolic trade-off where resources are redirected toward defense mechanisms. However, in compost tea-treated plants, particularly those affected by downy mildew, the decline in tartaric acid is less pronounced. This suggests that compost tea helps maintain a metabolic balance during infection, enabling the plant to continue producing essential metabolites like tartaric acid while simultaneously activating defense pathways. This stabilizing effect of compost tea under stress is supported by Pant et al. [38], who found that compost tea helps plants preserve metabolic activity while enhancing immune responses. The relatively higher levels of tartaric acid in compost tea-treated, infected leaves further support the idea that compost tea aids in maintaining grapevine health by fostering a more balanced metabolic response during pathogen attack. To fully unravel the intricate molecular underpinnings of this induced resistance, future investigations should explicitly focus on quantifying specific plant defense enzymes. Such analyses would provide invaluable mechanistic insights, moving beyond correlative metabolomic observations to directly demonstrate the activation of key enzymatic pathways. In summary, compost tea appears to promote beneficial microbial diversity, enhance defense-related metabolic pathways, and help sustain metabolic equilibrium under disease stress, making it a promising strategy for improving grapevine health and resilience to pathogens.

## 5. Conclusions

This study conclusively demonstrates that a repeated compost tea application, integrated with just two targeted cymoxanil treatments, represents a highly effective and sustainable strategy for managing grapevine downy mildew, even under severe disease pressure from climatically challenging conditions. Crucially, this innovative defense strategy achieved an over 80% reduction in synthetic fungicide use compared to conventional approaches, marking a significant advancement towards more environmentally conscious viticulture. Beyond its impressive field efficacy, our comprehensive investigation provides pivotal insights into the multifaceted mechanisms underlying compost tea’s suppressive power. We revealed its direct actions in limiting *P. viticola* infection and sporulation, thereby disrupting key phases of the pathogen’s life cycle. Furthermore, this research highlights compost tea’s profound ability to induce systemic resistance in grapevines, evidenced by the upregulation of defense-related metabolites like shikimic acid and the maintenance of essential compounds such as tartaric acid under stress. Concurrently, the repeated application of compost tea notably modifies the plant’s microbiome in both the rhizosphere and phyllosphere, enriching beneficial bacterial groups (e.g., *Pseudomonas*, *Sphingomonas*, *Bacillus*) that likely contribute to enhanced plant health and biocontrol potential. We also established that, with standardized feedstock and brewing, compost tea can exhibit a consistent and stable microbial composition, addressing a long-standing challenge in its practical application. While these promising results are specifically validated for the Montepulciano variety in the Puglia region, laying a strong foundation for sustainable viticulture, future research should broaden its scope. Investigating the efficacy of this integrated strategy across diverse viticultural contexts and grape varieties is essential for wider adoption. Additionally, further studies should evaluate the compatibility of compost tea with fungicides possessing different modes of action and explore its potential as a standalone or copper alternative in organic systems. Unraveling the precise molecular underpinnings of compost tea-induced resistance through the quantification of specific plant defense enzymes will further deepen our understanding and optimize its use.

## Figures and Tables

**Table 1:** Chemical characteristics of compost tea immediately before the application. Germination index values > 70 indicate the absence of phytotoxicity.

Parameters	Compost Tea
pH	6.4
EC (µS/cm)	1370
DOC (mg/L)	5123
BOD5 (mg/L)	1350
Germination index	108
Dissolved oxygen (mg/L)	4.6
Nitrate (mg/L)	1.4
Ammonia (mg/L)	7.2
Total Iron (mg/L)	2.3
Sulfates (mg/L)	170
Chlorines (mg/L)	298
Phosphates (mg/L)	18.4

## Data Availability

Reads of the sequence data have been deposited in the NCBI Sequence Read Archive (SRA) with accession n^o^: PRJNA1279629.

## Supplementary Materials

The following supporting information can be downloaded at https://www.mdpi.com/article/10.3390/jof11070527/s1, Table S1: The table presents the results of GC-MS analyses. Raw data were aligned and subjected to statistical analysis for each couple of conditions that were compared (Student’s *t*-test *p* < 0.05 and fold change > 2.0). Molecular features were identified by comparison of mass spectra with those stored in NIST20 library. “Match factor” indicates the similarity degree between the submitted spectra and the standard spectra. “*p*” denotes the *p*-value; “lnFC” is the natural logarithm of fold change; “ns” is not significant; “-” means that the metabolite was absent in one conditions and it was not possible to calculate *p*-value and fold change value. Significant decreases in metabolite abundance are highlighted in red, while significant increases are shown in blue.
