# Supplementary material for: Compost Tea Combined with Fungicides Modulates Grapevine Bacteriome and Metabolome to Suppress Downy Mildew
Source: J Fungi (Basel). 2025 Jul 16;11(7):527. doi: 10.3390/jof11070527 (PMC12298413; doi:10.3390/jof11070527)
Supplement: Supplementary file 1 [file jof-11-00527-s001.zip › jof-3673436-supplementary.pdf]

Supplementary materials

Table S1. The table presents the results of GC-MS analyses. Raw data were aligned and subjected to statistical analysis for each couple of conditions that were compared (Student's t-test p<0.05 and fold change>2.0). Molecular features were identified by comparison of mass spectra with those stored in NIST20 library. "Match factor" indicates the similarity degree between the submitted spectra and the standard spectra. "p" denotes the p-value; "lnFC" is the natural logarithm of fold change; "ns" is not significant; "-" means that the metabolite was absent in one conditions and it was not possible to calculate p-value and fold change value. Significant decreases in metabolite abundance are highlighted in red, while significant increases are shown in blue.

Compound	Match factor	Water inoculated vs Water		Compost tea inoculated vs Compost tea		Compost tea vs Water		Compost tea inoculated vs Water inoculated	
		p	lnFC	p	lnFC	p	lnFC	P	lnFC
**Glycerol**	927	ns	ns	ns	ns	ns	ns	Ns	ns
**Phosphate**	929	ns	ns	0,005953	-1,7303	ns	ns	Ns	ns
**Succinic acid**	932	ns	ns	0,013406	-1,4925	ns	ns	Ns	ns
**Glyceric acid**	905	ns	ns	3,19E-05	-3,0325	0,000113	3,0527	Ns	ns
**Erythronic acid lactone**	925	ns	ns	ns	ns	ns	ns	Ns	ns
**Malic acid**	904	ns	ns	ns	ns	ns	ns	Ns	ns
**Erythronic acid**	927	ns	ns	0,000559	-3,4016	0,00833	2,645	Ns	ns
**2-Hydroxyglutaric acid**	903	ns	ns	ns	ns	ns	ns	Ns	ns
**Xylonic acid lactone**	821	ns	ns	0,003455	-2,5199	0,005152	4,6412	Ns	ns
**Tartaric acid**	941	ns	ns	2,05E-06	-3,7803	1,23E-06	3,4376	Ns	ns
**Shikimic acid**	916	ns	ns	7,69E-05	-3,7237	0,002553	3,4758	0,006263	-2,8934
**Fructofuranose**	825	ns	ns	ns	ns	ns	ns	Ns	ns
**D-Fructose**	824	ns	ns	-	-	ns	ns	-	-
**Fructopyranose**	890	ns	ns	ns	ns	ns	ns	Ns	ns
**3-Hexenyl β-glucopyranoside**	836	ns	ns	0,024803	-2,4024	ns	ns	Ns	ns
**Talopyranose**	900	ns	ns	ns	ns	ns	ns	Ns	ns
**Gluconolactone**	840	ns	ns	0,017105	-4,6575	ns	ns	Ns	ns
**Ascorbic acid**	892	-	-	-	-	ns	ns	-	-
**Palmitic Acid**	944	ns	ns	ns	ns	ns	ns	Ns	ns
**D-Gluconic acid**	867	-	-	ns	ns	-	-	-	-
**Caffeic acid**	915	ns	ns	0,000614	-2,0288	ns	ns	0,013469	-1,95

**Phytol**	**937**	**ns**	**ns**	**0,021208**	**-1,2924**	**ns**	**ns**	**0,037741**	**-1,116**
**α-Linolenic acid**	**931**	**0,015639**	**-1,5819**	**0,017105**	**-1,5934**	**ns**	**ns**	**Ns**	**ns**
**Stearic acid**	**853**	**ns**	**ns**	**ns**	**ns**	**ns**	**ns**	**Ns**	**ns**
**Arachidic acid**	**816**	**ns**	**ns**	**ns**	**ns**	**ns**	**ns**	**Ns**	**ns**
**Sucrose**	**845**	**ns**	**ns**	**0,013791**	**-3,4926**	**0,012851**	**-3,1936**	**Ns**	**ns**
**Coutaric acid**	**903**	**ns**	**ns**	**0,000806**	**-4,163**	**2,44E-06**	**7,9292**	**Ns**	**ns**
**Tetracosanol**	**918**	**ns**	**ns**	**0,018906**	**-1,6704**	**ns**	**ns**	**Ns**	**ns**
**Chlorogenic acid methyl ester**	**853**	**ns**	**ns**	**0,003455**	**-2,6059**	**0,012797**	**2,1688**	**Ns**	**ns**
**Catechine**	**868**	**ns**	**ns**	**ns**	**ns**	**ns**	**ns**	**Ns**	**ns**
**1-Hexacosanol**	**888**	**ns**	**ns**	**0,013791**	**-2,3402**	**ns**	**ns**	**Ns**	**ns**
**1-Octacosanol**	**852**	**ns**	**ns**	**ns**	**ns**	**ns**	**ns**	**0,021797**	**2,0611**
**α-Tocopherol**	**933**	**ns**	**ns**	**ns**	**ns**	**0,033814**	**-1,4475**	**0,031086**	**-1,1527**
**Bracteatin**	**869**	**ns**	**ns**	**ns**	**ns**	**ns**	**ns**	**Ns**	**ns**
**Stigmasterol**	**868**	**ns**	**ns**	**ns**	**ns**	**ns**	**ns**	**Ns**	**ns**
**β-Sitosterol**	**943**	**ns**	**ns**	**0,015572**	**-1,2544**	**ns**	**ns**	**ns**	**ns**
